# Botulinum Toxin Injection in Acute Sixth Nerve Palsy in a 1-Year-Old Child: Case Report, Management Strategy, and Review of Literature

**DOI:** 10.22336/rjo.2025.05

**Published:** 2025

**Authors:** Corina-Ioana Merticariu, Mircea Merticariu, Mihaela Sorina Dragomir

**Affiliations:** 1 Department of Ophthalmology, “Dr. Victor Gomoiu” Children’s Hospital, Bucharest, Romania; 2 Department of Urology CF2 Hospital, Bucharest, Romania; 3 “Titu Maiorescu” University of Medicine, Bucharest, Romania

**Keywords:** sixth nerve palsy, botulinum toxin, acute onset strabismus, amblyopia, visual development, ocular motility disorders, child, CN VI = Cranial Nerve VI (Abducens nerve), BT = Botulinum Toxin, MRI = Magnetic Resonance Imaging, PD = Prism Diopters, ET = Esotropia, OS = Left Eye, IOP = Intraocular Pressure, VA = Visual Acuity

## Abstract

Sixth nerve palsy in children is a rare yet clinically significant condition that can result in strabismus and developmental complications. The acute onset of sixth nerve palsy can lead to esotropia, a sudden inward deviation of the eye due to impaired abduction. While most cases resolve spontaneously, botulinum toxin injection is gaining ground as a therapeutic intervention, particularly in pediatric patients with persistent or severe misalignment.

**Objective:**

To evaluate the effectiveness of botulinum toxin injection in managing acute sixth nerve palsy in a 1-year-old child with esotropia. The therapeutic outcomes and a review of current evidence are discussed, focusing on the efficacy, safety, and timing of botulinum toxin therapy in pediatric sixth nerve palsy. **Methods**: We report the case of a 1-year-old child diagnosed with acute sixth nerve palsy and treated with botulinum toxin injection to the medial rectus muscle. Follow-up assessments were conducted at 1 week, 3 weeks, and 8 weeks to monitor ocular alignment and visual development. **Results**: At the one-week follow-up, esotropia decreased from 50 to 20 prism diopters, with moderate upper eyelid ptosis. At eight weeks, ocular alignment was normal, with mild residual ptosis and improvement in amblyopia.

**Discussion:**

Sixth nerve palsy in children is uncommon but can arise from various underlying causes. In most cases, it resolves within a few months, but in severe or persistent cases, therapeutic interventions such as botulinum toxin injections can alleviate symptoms. The clinical case highlights that early intervention with botulinum toxin is a safe and effective method for improving ocular alignment.

**Conclusion:**

Botulinum toxin injection effectively improved ocular alignment and facilitated amblyopia recovery in this pediatric case of sixth nerve palsy, supporting its use as a safe adjunctive treatment in early intervention.

## Introduction

In children, sixth nerve palsy, also known as abducens nerve palsy, is a rare condition that presents significant clinical challenges. The abducens nerve (cranial nerve VI) innervates the lateral rectus muscle, which is responsible for the abducting of the eye. Damage to this nerve results in esotropia, typically accompanied by a limitation in the outward movement of the affected eye.

Although the condition is often self-limiting, especially in idiopathic cases, persistent or severe cases may benefit from therapeutic interventions, including botulinum toxin injections, which have emerged as an effective treatment modality in recent years [1,2].

## Methods

Sixth nerve palsy in children can be caused by a range of afflictions, from idiopathic or viral infections to trauma, tumors, vascular issues, and congenital disorders. Prompt diagnosis through clinical examination, neuroimaging, and appropriate testing is crucial for identifying the underlying etiology and guiding effective treatment. Treatment strategies, including observation, medical management, and, in some cases, botulinum toxin injection or surgery, are tailored to the cause and severity of the condition.

This study presents the case of a 1-year-old child diagnosed with acute sixth nerve palsy and treated with botulinum toxin and explores the broader implications of its use in pediatric cases.

## Results

While the vast majority of pediatric cases of sixth nerve palsy resolve within months without intervention, early therapeutic strategies, such as botulinum toxin, have been shown to provide rapid symptomatic relief, improve ocular alignment, and reduce the risk of amblyopia and long-term developmental visual issues [3].

## Case report

### Patient Presentation

A 1-year-old male infant was referred to the pediatric ophthalmology clinic with a sudden onset of inward deviation of the left eye (esotropia), which the parents had noted over the past 3 weeks. The patient had no history of trauma, fever, recent immunizations, or systemic illness. Developmentally, the child had reached age-appropriate milestones and had no significant past medical history. The parents were concerned about the new onset of strabismus, as the child had been otherwise healthy.

Upon clinical examination, the child exhibited a left esotropia of approximately 50 prism diopters at both distance and near, with severely impaired abduction of the left eye that could not reach the midline. There was a compensatory right head turn. No other ocular or systemic abnormalities were noted, and a neurological examination revealed no additional focal deficits. The deviation remained constant throughout the day. The child’s refraction was within normal limits (cycloplegic refraction OD +0.25/+1/156, OS +0.75/+0.50/146). Vision was age-appropriate in the right eye (VOD-fix and follow), however there was evidence of deprivation amblyopia in the left eye as the child strongly objected to covering the right eye and could neither fix nor follow with the left eye (VOS - does not maintain fixation, does not follow, opposes cover test - CT). The orbits and eyelids were symmetrical, with no narrowing of the interpalpebral fissure or retraction of the globe on attempted adduction of the left eye. Both the anterior pole exam and the fundus exam were unremarkable. The patient had previous photos that showed no preexisting ocular misalignment. A diagnosis of acute sixth nerve palsy was made based on the clinical presentation and the exclusion of other causes.

### Investigations

A comprehensive workup was performed to rule out any intracranial pathology. Magnetic resonance imaging (MRI) with contrast of the brain and orbits was obtained within a week to assess the integrity of the brainstem and rule out afflictions such as posterior fossa tumors, demyelination, or increased intracranial pressure. The MRI scan was unremarkable, with no evidence of structural abnormalities. Blood tests, including markers for systemic infections and inflammation, were regular. Given the acute onset and absence of other neurological deficits, a diagnosis of isolated acute sixth nerve palsy was confirmed.

### Management and Intervention

Given the acute onset of the strabismus and the severity of the deviation, as well as the child’s young age, a botulinum toxin injection was considered to address the misalignment and provide symptomatic relief while awaiting potential spontaneous recovery. A dose of 4 units (0.1 ml) of botulinum toxin A (Vistabel®) was injected into the medial rectus muscle of the left eye under general anesthesia. The procedure was performed under strict aseptic conditions, and the child tolerated the injection well without complications or allergic reactions.

### Follow-up and Outcome

**Fig. 1:**
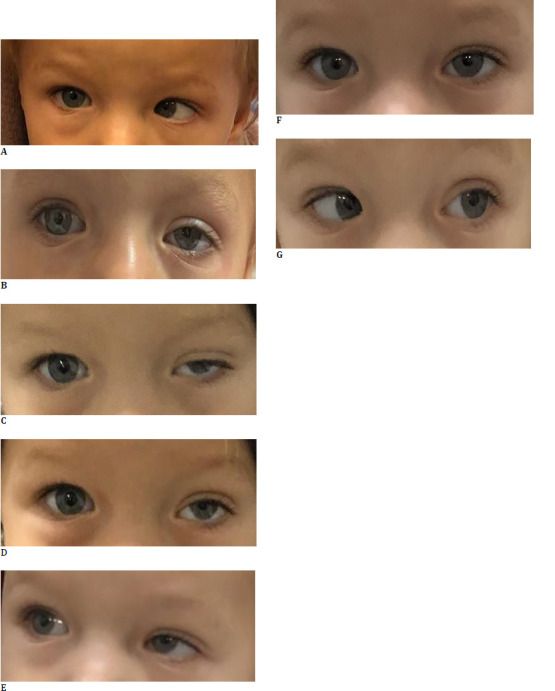
Changes in the patient’s ocular deviation following treatment (image published from the author’s archive, with consent of the patient’s parents). **A**. Large angle esotropia before Botuline Toxin Injection. LE ABD is not possible; **B-D**. Angle of deviation 1 week after Botulin toxin injection. **B**. Limited LE ADD; **C**. Significant Improvement of LE ET, moderate LE upper eyelid ptosis; **D**. Slight improvement in LE ABD, to the midline; **E-G**. Angle of deviation 8 weeks after Botulin Toxin injection. **E**. Nearly normal LE ADD; **F**. Ortotropia, mild LE upper eyelid ptosis; **G**. Nearly normal LE ABD

At the 1-week follow-up, the patient demonstrated significant improvement in the alignment of the left eye, with esotropia reduced to approximately 20 prism diopters and improved abduction toward the midline. Moderate ptosis was noted, with the left upper eyelid intermittently covering the pupillary reflex. By 8 weeks, the child exhibited normal ocular alignment, with near-normal ocular motility, significantly improved abduction, and a mild residual ptosis that did not require further intervention. The patient was monitored for any potential recurrences or long-term complications. Daily patching of the right eye was recommended throughout the follow-up to treat amblyopia. Improvement was noted as fixation could be maintained with the left eye, but objection to right eye occlusion persisted (**Fig. 1 A-G**).

## Discussion

### Epidemiology and Pathophysiology of Sixth Nerve Palsy in Children

Sixth nerve palsy in children is uncommon, with an estimated incidence of approximately 1 in 100,000 children per year [4]. It can be either congenital or acquired, with the latter most often occurring due to viral infections, trauma, or in some cases, vascular or neurological conditions [5]. In most children, sixth nerve palsy resolves within a few weeks to months, with spontaneous recovery of the nerve function [6]. However, in severe or persistent cases, therapeutic interventions such as botulinum toxin injections have been explored as a method to manage the condition and alleviate symptoms.

### Causes of Sixth Nerve Palsy in Children

Sixth nerve palsy (abducens nerve palsy) in children can arise from various underlying causes. The abducens nerve (cranial nerve VI) controls the lateral rectus muscle, which is responsible for the eye’s outward movement (abduction). When this nerve is impaired, the affected eye exhibits restricted abduction, resulting in a condition known as esotropia, characterized by an inward deviation of the eye. While sixth nerve palsy is rare in children, it can be associated with several factors, including congenital, acquired, and idiopathic conditions.

1. Idiopathic (Unknown Cause)

In many cases, the exact cause of sixth nerve palsy remains unknown, particularly in otherwise healthy children. Idiopathic sixth nerve palsy is often self-limiting, and the prognosis is generally good, with most children recovering within weeks to months [7]. However, long-term monitoring is required to rule out other underlying conditions.

2. Viral Infections

Viral infections are a significant cause of acquired sixth nerve palsy in children. These can involve the central nervous system (CNS) or directly affect the nerve. Common viral causes include:

- Varicella-Zoster Virus (VZV) can lead to viral neuritis or meningitis, resulting in nerve dysfunction;
- Epstein-Barr Virus (EBV) is known to cause cranial nerve palsies, including abducens nerve palsy;
- Influenza or other respiratory viruses can cause viral meningitis or encephalitis, leading to cranial nerve dysfunction;
- Coxsackievirus is associated with viral meningitis and occasionally affects cranial nerves.

3. Trauma

Head trauma, including concussion or cranial fractures, can cause direct damage to the abducens nerve. Damage may occur at the brainstem level or along the nerve path as it exits the brainstem. In cases of head injury, it is essential to assess for other associated injuries and neurological deficits. 4. Intracranial Space-Occupying Lesions

Brain tumors, cysts, or other space-occupying lesions in the brainstem or along the path of the sixth nerve can compress or damage the nerve. These may include:

- Brainstem tumors (e.g., gliomas or medulloblastomas) are one of the common tumors in children, with a peak age of onset between ages 5 and 8 years;
- Cerebellopontine angle tumors;
- Posterior fossa tumors (affecting the region where the sixth nerve originates) - more than 80% arise from the pons and can produce unilateral or bilateral abducens nerve palsy;
- Arachnoid cysts or epidermoid cysts.

When a child presents with sixth nerve palsy and signs of increased intracranial pressure (e.g., headache, vomiting, or altered consciousness), imaging such as MRI or CT is necessary to exclude a mass effect.

5. Vascular Causes

Vascular disease, including stroke or vascular malformations, can affect the abducens nerve. Vascular occlusion or ischemia can compromise the blood supply to the abducens nucleus or the nerve, leading to palsy. Common vascular causes include:

- Arteriovenous malformations (AVMs);
- Cavernous sinus thrombosis;
- Hypertension;
- Venous sinus thrombosis.

Vascular causes are more commonly seen in older children or those with underlying systemic conditions. 6. Intracranial Hypertension

Increased intracranial pressure (ICP) due to hydrocephalus, brain tumors, or other etiologies can cause cranial nerve palsies. Papilledema (swelling of the optic disc) and headache are commonly associated with increased intracranial pressure (ICP). In cases of elevated ICP, the sixth nerve is particularly vulnerable as it merges at the cerebellopontine angle and has a long intracranial course, making it susceptible to pressure at the brainstem level.

7. Inflammatory and Autoimmune Conditions

Inflammatory conditions, including autoimmune diseases, can result in sixth nerve palsy due to demyelination or direct nerve inflammation. Conditions to consider include:

- Multiple Sclerosis (MS). Though more common in adolescents, MS can cause demyelination of the sixth nerve or its nucleus in the brainstem.
- Systemic Lupus Erythematosus (SLE), Myasthenia gravis, Miller-Fisher syndrome, Thyroid eye disease. These autoimmune diseases can cause neurological symptoms, including cranial nerve palsies.
- Sarcoidosis. Known to cause granulomatous inflammation affecting the cranial nerves.
- Giant Cell Arteritis. Although rare in children, this condition can cause inflammation of the blood vessels that supply the cranial nerves.

8. Congenital Causes

In some cases, sixth nerve palsy is congenital and present from birth. This can be due to abnormalities in the development of the abducens nerve or its brainstem nuclei. Congenital sixth nerve palsy can present with a constant esotropia, and often, there is a history of abnormal eye alignment from an early age. It may be associated with:

- Duane Syndrome. A congenital ocular motility disorder where the abducens nerve is absent or maldeveloped, often presenting with limited eye movement and strabismus.
- Cranial nerve dysinnervation syndromes. Rare but possible.

9. Mitochondrial Disorders

Mitochondrial diseases such as Leber’s hereditary optic neuropathy (LHON) and Kearns-Sayre syndrome can sometimes present with cranial nerve palsies, including the sixth nerve. These conditions may be associated with a variety of systemic findings, including muscle weakness, visual impairment, and hearing loss.

10. Neurodegenerative Diseases

Certain neurodegenerative diseases, although rare in children, can involve cranial nerves and lead to sixth nerve palsy. Spinocerebellar ataxia or Leigh syndrome can involve the brainstem and affect the abducens nerve.

11. Infection-Related Causes

Infections such as bacterial meningitis or tuberculous meningitis, sphenoiditis, or lateral recuts myositis can cause inflammation or compression of the cranial nerves. Tuberculous meningitis should be considered in children from endemic areas, particularly in the presence of other neurological symptoms.

### Botulinum Toxin in Strabismus Management

Botulinum toxin has been used for decades in the treatment of adult strabismus. Still, its use in pediatric strabismus has gained attention over the last two decades due to its ability to relieve ocular misalignment without surgical intervention [8] temporarily. In the case of sixth nerve palsy, botulinum toxin causes temporary paralysis of the medial rectus muscle, which is responsible for the inward deviation of the eye. This allows the lateral rectus muscle (innervated by the unopposed abducens nerve) to gain a greater functional capacity, thus improving ocular alignment and reducing the severity of esotropia [7].

Several studies have demonstrated that botulinum toxin is particularly effective in treating acute or subacute cases of sixth nerve palsy in children, as well as in preventing contracture of the medial rectus muscle. It, therefore, provides rapid symptom relief and improvement in ocular alignment while the nerve recovers. A study by Hatt et al. [9] found that botulinum toxin significantly reduced the degree of strabismus in pediatric patients with acute or chronic sixth nerve palsy. Similarly, Meier et al. [10] highlighted that botulinum toxin is a safe and effective alternative to surgical correction in children under 2 years of age, as it avoids the risks associated with general anesthesia and surgery.

• Timing and Dosage

The timing of botulinum toxin administration is critical in optimizing its efficacy. The ideal window for injection is typically within the first 6 weeks of symptom onset, after which the risk of irreversible ocular misalignment or amblyopia increases [11]. The appropriate dosage varies depending on the severity of the misalignment and the patient’s age, with most pediatric cases requiring between 2.5 and 5 units of botulinum toxin per muscle [12]. In our case, where four units were administered according to the recommended dose for a 1-year-old child, the outcome was satisfactory.

• Safety and Complications

Botulinum toxin is generally well-tolerated in pediatric patients, with minimal risk of adverse effects when administered by experienced clinicians. The most common side effects include transient ptosis, transient diplopia, or mild conjunctival irritation. Serious complications are rare but can consist of systemic effects such as dysphagia, respiratory distress, or anaphylaxis [13]. Long-term follow-up is essential to monitor for any recurrence of misalignment or development of amblyopia.

## Conclusion

Although rare, acute sixth nerve palsy in children poses significant challenges in terms of diagnosis and management. Botulinum toxin injection is a promising treatment for improving ocular alignment, particularly in cases with substantial or persistent strabismus. This case demonstrates that early intervention with botulinum toxin can lead to rapid and sustained improvement in ocular alignment and recovery from amblyopia, thereby promoting normal visual development in a 1-year-old infant. Further research is needed to determine the optimal timing, dosage, and long-term outcomes of botulinum toxin therapy in pediatric sixth nerve palsy.
